# Hybrid repair of the complex celiac artery aneurysm

**DOI:** 10.1016/j.ijscr.2025.111413

**Published:** 2025-05-08

**Authors:** Kwangjin Lee, Sungsin Cho, Jin Hyun Joh

**Affiliations:** a Department of Surgery, Kangwon National University Hospital, Kangwon National University School of Medicine, Chuncheon, Republic of Korea; b Department of Surgery, Kyung Hee University Hospital at Gangdong, Kyung Hee University School of Medicine, Seoul, Republic of Korea

**Keywords:** Celiac artery aneurysm, Hybrid surgery, Open surgery, Endovascular repair, Case report

## Abstract

**Introduction and importance:**

Celiac artery aneurysms (CAAs) are rare but potentially life-threatening vascular abnormalities with high mortality rates if left untreated. While open surgical repair has been the standard treatment, it carries significant risks, particularly in elderly or comorbid patients. Endovascular surgery offers a less invasive alternative; however, anatomical complexities can limit its effectiveness. Herein, we report the hybrid repair of a complex CAA case.

**Case presentation:**

The patient was an 82-year-old male who had a celiac aneurysm that increased in size during follow-up and was accompanied by a pseudoaneurysm. The first endovascular repair through the right common femoral artery failed because of an anatomic barrier. A week later, a second endovascular repair was attempted using an open approach, followed by covered stent deployment and branch embolization through the common hepatic artery. Completion angiograms demonstrated no endoleaks, successful celiac branch embolization, and patent common hepatic and splenic arteries. At follow-up after a week, the patient was stable and showed a patent-covered stent and complete aneurysm exclusion.

**Clinical discussion:**

This case highlights the value of utilizing hybrid approaches when conventional endovascular methods are limited by anatomical challenges, particularly in high-risk patients where traditional open repair may carry an increased morbidity risk.

**Conclusion:**

This case report describes a successful hybrid approach for the repair of a complex CAA. The hybrid approach, combining open surgical access with endovascular techniques, allowed for complete aneurysm exclusion and maintained the patency of the hepatic and splenic arteries. Hybrid repair can be a safer alternative to purely endovascular or open surgical approaches in complex CAA cases.

## Highlights

• Hybrid repair was successfully performed for a complex celiac artery aneurysm.
• Initial endovascular repair failed due to anatomic barriers, necessitating a hybrid approach.
• The procedure involved open surgical access, covered stent deployment, and branch embolization.
• Completion angiograms confirmed aneurysm exclusion and patent hepatic and splenic arteries.
• This case highlights hybrid repair as a viable alternative for anatomically challenging CAAs.

## 1 Introduction

A celiac artery aneurysm (CAA) is a rare vascular abnormality that accounts for only 0.005–0.01 % of all aneurysms [1]. Most CAAs are asymptomatic and detected incidentally, but their risk of rupture poses a significant threat to patient survival, with mortality rates reaching up to 40 % in untreated cases [2]. Conventional open surgical approaches, such as aneurysmorrhaphy and bypass techniques, have long been the standard of care [3]. However, these methods carry significant risks, including high morbidity and mortality, particularly in elderly patients and those with comorbidities. In recent years, endovascular repair has emerged as a less invasive alternative, offering shorter recovery times and reduced perioperative risks [[4], [5], [6], [7], [8]]. Despite these advantages, endovascular repair has limitations, especially in cases with complex anatomy or unfavorable vascular configurations. Hybrid repair, which combines open surgical access and endovascular techniques, has shown promise in overcoming these challenges [9,10]. Herein, we report the successful hybrid repair of a complex CAA case. This article has been reported according to the SCARE criteria [12].

## 2 Case report

**Fig. 1:**
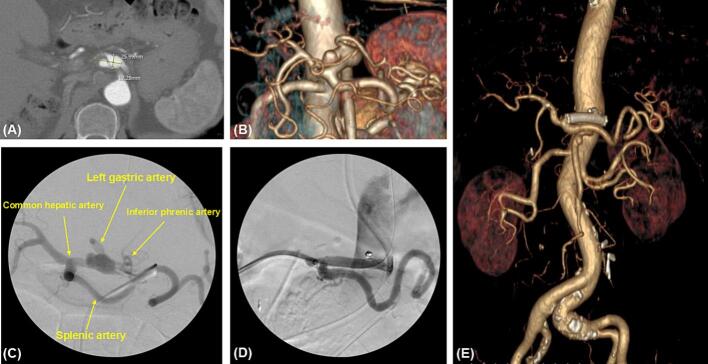
Preoperative, intraoperative, and postoperative images of the patient. (A) The preoperative axial view of the computed tomography scan showed that the size was approximately 26 mm in width and 12 mm in height. (B) The volume-rendering image of preoperative computed tomography showed the saccular shaped pseudoaneurysm in the celiac artery. (C) Intraoperative contrast angiogram showed the aneurysm of celiac artery on the first operation. (D) Completion angiogram after hybrid surgery showed the complete resolution of aneurysm and no endoleak. (E) Postoperative volume-rendering view confirmed that the aneurysm was we completely excluded without an endoleak, and the flow of the common hepatic artery and splenic artery showed good patency.

The patient was an 82-year-old male patient with no specific underlying medical disease but with a history of laparoscopic anterior resection for sigmoid colon cancer and subsequent wedge resection of the right upper lobe for lung metastasis. During follow-up after chemotherapy, an 11 mm CAA was found on computed tomography of the abdomen and pelvis. The patient showed no CAA-related symptoms. During the 2-year follow-up period, the size increased to 12 × 26 mm (Fig. 1A). The aneurysm started 2 cm distal to the origin of the celiac artery, had an eccentric shape, a length of 26 mm, and a diameter of 12 mm. The left gastric artery branched above the aneurysm, and a pseudoaneurysm with a thrombus was observed on the posterolateral side. After the aneurysm, the celiac artery runs approximately 1 cm and is divided into the common hepatic and splenic arteries (Fig. 1A, B). Despite the absence of symptoms, the risk of rupture was high due to the size (26 mm) and the presence of a pseudoaneurysm. Therefore, treatment was decided upon.

First, endovascular repair was performed using access through the right femoral artery, and celiac artery selection was successful. Despite the successful advancement of the angiographic catheter to the lesion, the guiding sheath could not pass through the vessel, even with the use of a stiff wire, as the celiac artery originated obliquely. Considering the potential risk of vessel injury or rupture, we decided to terminate the procedure. Therefore, after obtaining only the angiogram, the procedure was stopped, and a second operation was planned (Fig. 1C).

**Fig. 2:**
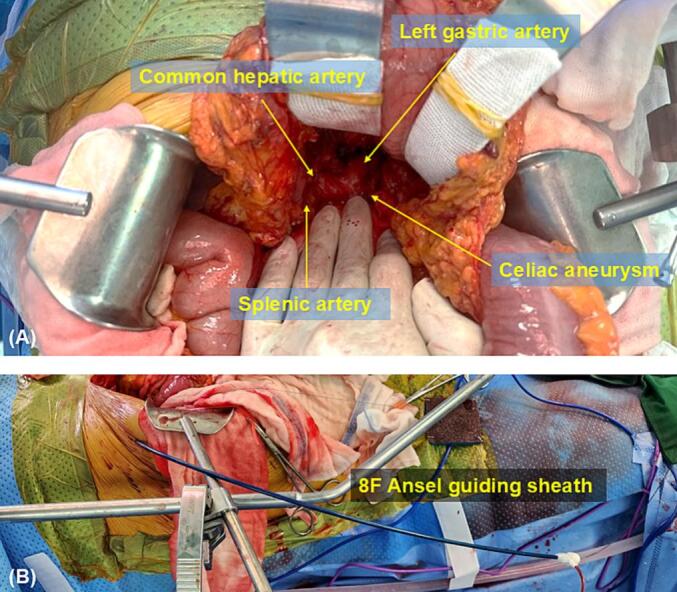
Surgical field during hybrid repair of celiac artery aneurysm. (A) After opening the lesser sac, the operative field showed the main branches of the celiac trunk and saccular shaped pseudoaneurysm. (B) An 8F Ansel guiding sheath was inserted into common hepatic artery through the abdominal wall of right flank.

A week later, a second surgery was performed. After the midline incision, the gastrocolic ligament was incised, and the lesser sac was opened, exposing the celiac aneurysm, the left gastric artery, and the common hepatic artery (Fig. 2A). The left gastric artery was ligated to prevent an endoleak. After retrograde puncture in the middle of the common hepatic artery, an 8F Ansel guiding sheath was inserted using the Seldinger technique, and the Ansel guiding sheath was fixed on the abdominal wall (Fig. 2B). The inferior phrenic artery was then embolized with a 3 mm × 6 mm coil (Interlock; Boston Scientific, Natick, MA, USA). For the aneurysm, a 6 mm × 40 mm covered stent (Covera Plus; Bard, Tempe, AZ, USA) was inserted using the proximal landing zone immediately before the splenic artery. A completion angiogram demonstrated the satisfactory exclusion of the aneurysm and patent graft, with brisk flow into the common hepatic and splenic arteries (Fig. 1D).

The patient underwent follow-up Computed Tomography Angiography (CTA) 1 week after surgery. The aneurysm was completely excluded without endoleaks, and the common hepatic and splenic arteries remained patent (Fig. 1E). At the 2-year follow-up CTA, stent patency was well maintained with no complications.

## 3 Discussion

Celiac artery aneurysms are rare but clinically significant because of their potential for rupture and associated high mortality rates. Untreated CAAs have reported rupture rates of 7 %, with mortality rates reaching 25–70 % in ruptured cases, compared to 5 % in elective repair [2,3]. Recently, CAAs have been diagnosed with increasing frequency using advanced imaging techniques. According to the guidelines released by the Society for Vascular Surgery, the treatment indication can be a true aneurysm >2 cm with a demonstrable increase in size, pseudoaneurysms of any size, or associated CAA-related symptoms [11].

In our case, the patient was undergoing adjuvant chemotherapy for sigmoid colon cancer when the CAA was incidentally discovered. While some studies have reported associations between chemotherapy and the growth of aortic aneurysms [13,14], recent literature specifically examining visceral artery aneurysms, including celiac aneurysms, suggests different findings. Becker von Rose et al. [15] reported that cancer therapy did not significantly influence the annual growth rates of visceral artery aneurysms, with average growth rates comparable to those of non-cancer patients. In our patient, although the aneurysm increased in size during follow-up, the causal relationship between chemotherapy and progression of the celiac artery aneurysm was unclear and warrants further investigation.

Effective management of CAAs often depends on their size and anatomical complexity. Open surgical repair has long been considered the gold standard for CAA treatment, offering definitive resolution of the aneurysm and durable long-term outcomes [[1], [2], [3]]. However, these procedures require extensive dissection and aortic clamping, which are invasive and can cause significant morbidity, particularly in elderly patients and those with comorbidities.

Endovascular techniques such as stent grafting and coil embolization have emerged as less invasive alternatives, demonstrating promising results in reducing perioperative risks and recovery times. These techniques are especially advantageous for elderly patients and those for whom open surgery is deemed unsuitable [5,7]. Nevertheless, anatomical challenges such as branch vessel involvement and vessel tortuosity can complicate purely endovascular approaches, sometimes leading to procedural failure or suboptimal outcomes [6,8].

Hybrid approaches, which combine open surgical access with endovascular techniques, have shown increasing utility in the management of anatomically complex CAAs. Although reports of hybrid repair specifically for CAAs are limited, hybrid approaches have been increasingly applied to a variety of vascular anomalies, such as peripheral artery occlusive disease and thoracoabdominal aortic aneurysms. These procedures have demonstrated high technical success rates and are considered effective alternatives in selected cases [16,17]. Case reports and small series suggest that hybrid techniques can address the limitations of purely endovascular approaches by enabling precise access and control in challenging anatomical scenarios [9,10]. While promising, its long-term durability, particularly with covered stents, requires further investigation [18]. Additionally, these approaches require specialized expertise and resources, limiting their availability to high-volume centers.

Despite advancements, the long-term outcomes of hybrid repair and covered stents remain under-reported. Longitudinal studies are needed to evaluate the durability of these interventions [12]. Sharing detailed case reports, such as ours, contributes to the growing body of evidence supporting hybrid techniques as a valuable addition to the therapeutic arsenal for CAA management.

## 4 Conclusion

This case report details a successful hybrid approach to repairing a complex CAA in an 82-year-old male patient in whom initial endovascular intervention failed due to anatomical challenges. The hybrid approach, combining open surgical access with endovascular techniques, allowed for complete aneurysm exclusion and maintained the patency of the hepatic and splenic arteries. This report highlights the value of hybrid repair in complex CAA cases as a safer alternative to purely endovascular or open surgical approaches.

## Author contribution

Kwangjin Lee: Study concept and design, drafting of the manuscript, writing, and critical revision of the manuscript.

Sungsin Cho: Literature review.

Jin Hyun Joh: Supervision, critical revision, and final approval of the manuscript.

## Consent

Written informed consent was obtained from the patient for the publication of this case report and the accompanying images. A copy of the written consent is available for review by the Editor-in-Chief of this journal upon request.

## Ethical approval

Ethical approval was not applicable to this study, as our institution's IRB committee at Kangwon National University does not mandate approval for reporting individual cases or case series.

## Guarantor

Jin Hyun Joh.

## Research registration number

Not applicable to this case report.

## Funding

This research did not receive any specific grants from funding agencies in the public, commercial, or not-for-profit sectors.

## Conflict of interest statement

The authors have no conflicts of interest to declare.
